# A Review of Pain Assessment in Pigs

**DOI:** 10.3389/fvets.2016.00108

**Published:** 2016-11-28

**Authors:** Sarah H. Ison, R. Eddie Clutton, Pierpaolo Di Giminiani, Kenneth M. D. Rutherford

**Affiliations:** ^1^Animal Behaviour and Welfare, Animal and Veterinary Sciences, Scotland’s Rural College (SRUC), Edinburgh, UK; ^2^Easter Bush Veterinary Centre, Royal (Dick) School of Veterinary Studies, The University of Edinburgh, Midlothian, UK; ^3^Food and Rural Development, School of Agriculture, Newcastle University, Newcastle upon Tyne, UK

**Keywords:** pig, pain, pain assessment, welfare, review

## Abstract

There is a moral obligation to minimize pain in pigs used for human benefit. In livestock production, pigs experience pain caused by management procedures, e.g., castration and tail docking, injuries from fighting or poor housing conditions, “management diseases” like mastitis or streptococcal meningitis, and at parturition. Pigs used in biomedical research undergo procedures that are regarded as painful in humans, but do not receive similar levels of analgesia, and pet pigs also experience potentially painful conditions. In all contexts, accurate pain assessment is a prerequisite in (a) the estimation of the welfare consequences of noxious interventions and (b) the development of more effective pain mitigation strategies. This narrative review identifies the sources of pain in pigs, discusses the various assessment measures currently available, and proposes directions for future investigation.

## Introduction

Recent reviews have focused on the assessment and alleviation of pain in management procedures in pigs (1–3). A further review discussed the completeness of reporting on outcomes in pain mitigation studies involving neonatal piglets undergoing painful management procedures (4). Another discussed the reporting of pain management (or lack of) in pigs used in experimental surgery (5). The recognition and treatment of pain in pet pigs has also been reviewed (6).

This review focuses on the assessment of both “experimental” and “naturally occurring” pain, drawing examples from the farm animal and biomedical literature. The aim is to provide a basis for the future study of pain and pain assessment in pigs and to create tools for improving pig welfare.

### The Multidimensional Pain Experience

Any pain assessment method must recognize that the pain experience – at least in humans – has three conceptual components, which, nevertheless, may be differentiated by brain imaging studies (7): sensory-discriminative, affective-motivational, and cognitive-evaluative, which interact with each other to form the pain experience (8). Cervero (9) described the sensory component as the detection and processing of noxious stimuli including reflex responses. The affective-motivational component is the aversive response or the “*unpleasantness*” of pain, and the cognitive component includes the worry about what the pain is, what it means, and how will it affect the future (9).

As pain is a subjective, personal experience, attempts to objectively measure it scientifically in animals are particularly challenging. Verbal self-report is considered the “gold standard” for pain assessment in humans, but even verbal pain communication has limitations (10). The International Association for the Study of Pain (IASP) state that an inability to communicate verbally does not negate the ability to experience pain, and the need for pain relief, and makes the ability to assess pain using non-verbal methods particularly important (11, 12).

### Pain in Animals

Humans and vertebrates share similar neuroanatomical structures associated with pain perception, i.e., nociceptors, nociceptive pathways, and processing areas in the central nervous system (CNS) (13, 14), which justifies their extensive use in the study of human pain (15). Demonstrating that an animal is able to detect a noxious stimulus is straightforward and for many years, since the first methodology was described (16), scientists have used quantitative sensory testing (QST) to measure the thresholds at which animals respond. This simple, functional avoidance response is a basic ability and present, even in amebae. However, it does not encompass the complex nature of clinically relevant pain (9).

The ability to demonstrate that an animal perceives a painful experience as unpleasant or aversive is considerably more challenging. Nevertheless, many animals exhibit complex behavioral and physiological responses indicative of pain appreciation in situations where pain would be present in humans, and which are ameliorated by analgesics [e.g., Ref. (14)]. More recent behavioral paradigms have been developed to demonstrate the affective-motivational component of pain [e.g., Ref. (17)], but the ability of animals to experience the cognitive component of pain remains controversial (9).

### Pain Assessment

Effective pain assessment tools should meet the requirements of the COnsensus-based Standards for the selection of health Measurement INstruments (COSMIN) taxonomy (18), and at least, be valid, reliable, and sensitive. Validity ensures the pain assessment device is measuring pain and not a related state such as anxiety, fear, or general sickness behavior. Reliability is tested to ensure the assessment tool is repeatable between (inter) and within (intra) observers and is not influenced by observer bias or observer subjectivity. Pain assessment tools should also be sensitive, i.e., be able to detect pain at low levels and increase linearly with the severity of pain.

Validation can be formally assessed against a “gold standard” measure (19). However, since there is no “gold standard” method of animal pain assessment; indicators must be thoroughly assessed using experimental approaches to ensure validity. Behavioral and physiological measures interpreted as indicative of pain in pigs have been investigated in experimental settings by (i) observing individuals before, during, and after a painful intervention or event; (ii) comparing individuals thought to be in pain to controls (no procedure, left undisturbed) or shams (no procedure but handled as if undergoing one); (iii) observing individuals with and without analgesia or analgesia delivered at different doses; and (iv) observing whether indicators of pain increase with the severity of pain. Ideally, a combination of these methods would be used to test the validity of pain assessment measures.

The ideal pain assessment tool should be well-validated. It would correctly differentiate animals in pain from those that are handled only or are left undisturbed. Its output would decrease or disappear with the use of effective analgesics compared with a placebo, and in a dose-dependent fashion. In addition, it would show a linear increase with rising pain intensity and would not “plateau” or show a “ceiling” effect. It would be specific to pain and not related to other features of the animals’ welfare state, such as fear, inappetence, immunosuppression, impaired mobility, or other sickness behaviors (though these are additionally relevant for assessing the overall level of impaired welfare or suffering in relation to injury or disease). For clinical use, it should be readily applied, inexpensive, non-invasive, provide immediate (not retrospective) information, and be repeatable over time (intraobserver reliability) and between observers (interobserver reliability). The ideal pain assessment device would equally appraise both sensory and emotional response to pain, i.e., measure what the pain means to the animal, rather than simply quantify the magnitude of noxious stimulation.

## Sources of Pain in Pigs

### Management Practices

Pigs undergo painful procedures on farms, e.g., tail docking, teeth resection, castration, ear tagging, or notching (1) to enable identification, prevent injurious behavior, improve ease of management, and increase product quality (20). They are also injected with iron supplements, vaccines, and drugs, when necessary. The frequency with which these procedures are conducted on the farmed pig population has provided the opportunity to study acute and chronic pain responses and pain reduction strategies (21).

Tail docking (partial tail removal using clippers, cautery irons, knives, scalpels, or scissors) aims to reduce the incidence of, and damage caused by tail biting (22). Surgical castration entails scrotal incision, the blunt dissection of testicular from surrounding fascial tissue, and hyper-extension of the spermatic cord until it spontaneously ruptures or is cut (23) usually without anesthetic. The procedure aims to reduce or eliminate “boar taint,” and undesirable sexual behavior; castrated males are considered less aggressive and easier to manage (24). Teeth resection – the variable removal of needle (canine) teeth with cutting or grinding devices – is done to minimize bite injuries to littermates and the sow’s udder during the competition for functional teats (25). Ear notches or tags, tattoos, or injected transponders enable carcass identification, which is a prerequisite in meat quality improvement and public health schemes (26). All these management procedures induce tissue damage, which can potentially lead to a greater risk of infections and consequent pain.

### Lameness

Locomotor pain in pigs is indicated by lameness, by carrying a foot, favoring a leg, or being unable to get up and move around (27). Lameness is an important source of pain in terms of prevalence and severity (28), is ranked as the most important measure of pig welfare assessment in one survey of experts (29), and is thought to affect 10–20% of pigs on commercial farms (30). In a recent survey in the UK, lameness with minimal weight bearing in pigs was given a high score for pain by farmers (6.3 out of the 10) and veterinarians (7.0 out of the 10) (31). It is also a global concern in terms of economic loss, as locomotor problems are a common reason for early culling of sows, on-farm euthanasia, contribute to veterinary treatments costs, and carcass downgrading (32, 33). Lameness can be caused by injury due to poor housing conditions, non-infectious and infectious conditions, and degenerative diseases (34–36). Individuals can also be genetically predisposed to lameness (37), and lameness can be associated with inadequate nutrition, relating to growth rate (38). Additionally, a chemically induced transient lameness model was developed in sows to study clinical lameness detection and analgesic strategies (39).

### Parturition

The presence of anatomical and physiological structures associated with pain, as well as increasing experimental evidence, means it is generally accepted that a number of mammalian species experience pain at parturition (14, 40–42). Parturition pain originates from uterine contractions, fetal expulsion, and inflammation of the uterine tract (43). After birth, pain in human females results from intermittent uterine contractions during involution, when the uterus returns to its normal size and from tissue damage associated with birth (44). Evidence suggests pigs experience post-farrowing pain, as putative behavioral indicators of pain continue beyond the expulsion phase of farrowing in sows (45). Pain is greater during dystocia (46), but this is estimated to occur in only 0.25–1.0% of commercial sow farrowings (47), probably because the porcine fetus is small relative to the mother. In a survey of UK farmers and veterinarians, using a 11-point pain scale, respondents, respectively, awarded median scores of 3.8 and 4.5, for uncomplicated farrowings, and greater scores (6.7 and 7.3) to difficult farrowings in which an internal examination was required (31). This is important, given that farmer respondents to the survey considered 5.3% of gilts and 3.7% of sows to have difficulty farrowing and regularly used injectable oxytocin products to increase the frequency and intensity of uterine contractions, which are themselves associated with pain (48).

### Disease

Diseases (49) including mastitis (34) and streptococcal meningitis (35) cause pain in pigs. Using the aforementioned pain scale, farmers and veterinarians awarded scores of 7.5 and 7.3, respectively, for infectious mastitis. In contrast, respiratory diseases were given moderate scores of 5.1 (by both farmers and veterinarians), while gastrointestinal diseases were also given moderate scores by farmers (5.6) and veterinarians (4.5) (31). Disease can result from poor climatic conditions, poor hygiene, inappropriate housing conditions, and genetics (49). Accurate information on the overall prevalence of disease in commercial pigs is lacking. One study investigating the clinical signs of disease in Danish finisher pigs provide some indication on the level of certain types of disease on-farm (50), and another Danish study provides information on the level of diarrhea and intestinal pathogens in pigs (51). Abattoir data is one method that has been used to estimate disease prevalence (52, 53) but is likely to underestimate the problem, since many diseased pigs may not make it to slaughter. In general, due to the nature of intensive pig farming, close contact between individual’s results in rapid transmission of disease within and between pig herds, and several endemic diseases are a feature of modern pork production (54). Therefore, as on-farm disease problems are widespread, painful disease states are a significant source of pain for a large number of pigs.

### Injuries

Pigs receive injuries when fighting, mounting, being handled ineptly, or reared under suboptimal conditions. Severe injuries like fractures and muscle tearing cause considerable pain, and leg fractures were awarded high scores by farmers (8.8) and veterinarians (9.5) (31). Other injuries include teat damage from biting piglets, cuts, bruises, and vulvar damage from aggressive interaction, while ear-, flank- and tail-biting occur under conditions of poor housing or environmental conditions (34). Shoulder ulcers are common in sows and are connected to many factors [for a review, see Ref. (55)] and were scored as moderately painful by farmers (5.6 out of the 10) and veterinarians (5.6 out of the 10) (31). The accurate prevalence of injury in commercial pigs is also unclear. Injury is another widespread problem associated with modern pork production (54) and was the most observed clinical sign observed in Danish finisher pigs (50). The prevalence of foot and limb lesions in preweaning piglets on commercial farms in Ireland ranged from 0 to 100% (56), and the overall prevalence on commercially representative pig farms in England was 39.6% (57). One study showed a high prevalence of lesions on the limbs (93%), body (20%) (58), and feet (59) of commercial sows in England. In addition, hernias are a relatively common condition in pigs (60), with umbilical hernias shown to affect 0.78% of finisher pigs in Denmark (50), and in severe cases, hernias can become ulcerated (61). Gastric ulceration is another management-related condition and potential source of pain in pigs, with one recent study in the UK showing 6% severe ulceration and 73% mild ulceration at slaughter (62).

### Biomedical Research

The extensive use of pigs in biomedical research is justified on the basis of their physiological and anatomical similarity to humans, their large body size facilitates the collection of multiple samples, and they are omnivores with similar digestive systems to humans (63). Evidence-based information on postoperative pain management in pigs is lacking, despite the greater availability of analgesic drugs compared with farmed pigs (5). Pigs bred specifically for laboratory use, e.g., the Göttingen mini-pig, tends to be less vocal when manually restrained than commercial pigs. This may be the result of selective breeding for placidity or size and should not be taken to indicate an absence of pain or distress.

## Pain Assessment in Pigs

### Behavior

#### Pain-Related Behavior

Spontaneously occurring behaviors arising or increasing in the context of painful conditions have been quantified in several studies, including observations of pigs before, during, and after painful events (39, 45, 64, 65), comparisons of those in pain with controls or shams (66–74), with and without analgesia or anesthesia (71, 75–82), and with varying degrees of severity (39, 65).

An early study of 4-week-old piglets identified “castration-specific” behaviors including trembling, leg shaking (jerking a hind leg back without scratching), sliding (sits and slides along on hind quarters), and tail jerking (64). Another study observed pain-specific behaviors after tail docking (tail wagging and jamming), teeth clipping (teeth champing), and ear notching (head shaking) (66). Other studies have observed more tail wagging, tail jamming, and bottom scooting (sitting, dragging bottom along the floor) in piglets following tail docking (67–69), and tail wagging associated with castration (70). In sows, shoulder rubbing has been associated with shoulder sores (73) and trembling, tail flicking, pulling the back leg forward, and pawing are putative pain indicators at parturition (45). Two studies showed no difference in “pain-related” behavior between individuals undergoing procedures and sham individuals for castration (71) and tail docking (72). This may have arisen because the scan sampling used may have missed relevant behaviors. Alternatively, there may have been fewer pain behaviors expressed because the observer was present. Both these issues have been discussed in relation to the use of spontaneous pain-related behavior in rodent pain assays (83).

Other pain-related behavior associated with castration include huddling up (lying with at least three legs tucked under body), spasms (quick, involuntary muscle contractions), stiffness (lying with extended, tensed legs), prostration (sit or stand motionless, head down), trembling (shivering as if cold), and rump scratching. Pre-castration treatment with the non-steroidal anti-inflammatory drug (NSAID) meloxicam resulted in less pain-related behavior afterward (75), irrespective of whether a local anesthetic was used. In another study, fewer piglets in a meloxicam-treated group exhibited pain-related behavior (78). As expected, little or no difference in pain-related behavior was seen after castration performed with and without general anesthesia (71, 76, 77). This is expected, given the use of anesthetic during the procedure, but no post-procedural analgesia. In addition, no difference in bottom scooting or huddling was seen following castration when long- or short-acting topical local anesthetics were applied to the severed spermatic cords and scrotal incisions (71).

Recent pig studies have measured leg loading and movement using pressure mats, force plates motion-capture systems, and accelerometers as objective measures of locomotor abnormalities associated with lameness. Among other things, lame individuals show asymmetrical weight distribution between legs, increased step frequency, stance time, and altered stride length (74, 84, 85). The opioid analgesic buprenorphine decreased pressure mat gait asymmetry (79), and the NSAID meloxicam decreased step frequency and produced better symmetry of pelvic limb movement (82) in pigs with naturally occurring lameness. In a chemically induced transient sow lameness model, leg loading and movement improved with the use of the NSAIDs meloxicam and flunixin (80), while the severity of lameness decreased and resolved by day 7 following induction (39, 65). However, another study using the same lameness model found no improvement with the use of the NSAIDs sodium salicylate or flunixin (81). The authors of the more recent study (80) suggested this difference could be due to the use of additional time-points compared with the previous study (81), which better coincided with when the drug is thought to be most effective. They also suggested that using more sows in the study created greater statistical power (80). In a more recent study, continuous behavioral observations, but not scan sampling, detected differences in lame sows treated with flunixin, whereas both techniques detected improvements to sows treated with meloxicam, in this case, demonstrating the greater efficacy of meloxicam over flunixin to treat lameness in sows (86).

#### Escape Behavior

Escape or avoidance behavior has been used as a measure of pain recorded during a pre- and post-procedural handling period (87), compared individuals undergoing painful and sham procedures (88), and those given a local anesthetic or not (87, 89). These behaviors, scored in terms of the frequency (75, 76, 88), duration (87), and/or intensity (76, 87, 89) of resistance movements have also been recorded during different periods of the castration procedure (89), and when different techniques were used (88), as these are likely to affect the severity of pain experienced. Duration and intensity scores of both defense behavior increased from the pretreatment to treatment period in piglets castrated without anesthetic, whereas they did not differ in sham-castrated piglets (87). When procedural time was taken into account, no differences in escape attempts and leg kicks were found between different methods of teeth resection, tail docking, identification, iron administration, and sham procedures (88). Not unexpectedly, a significant reduction in escape behavior during castration was exhibited in piglets given a local anesthetic (75, 87). These measures are useful in assessing pain associated with severe acute events, like tail docking or castration, in order to evaluate pain reduction strategies, such as the use of local anesthetics.

#### Posture and Posture Changes

Posture and posture changes have been measured before, during, and after painful events (64, 90), compared with controls/shams (68, 69, 73, 91–94), with and without anesthesia or analgesia (75, 76, 82, 95–98) and with the severity of painful conditions (99).

Docked piglets tended to spend more time sitting than shams following the procedure, which may relieve pain at the tail while protecting the wound site (68). Wemelsfelder and Van Putten (64) reported longer latencies to lie down in piglets 1 day post-castration at each individual phase of the lying sequence. In addition, the lying sequence differed significantly. Another study described changes in posture after castration, which included weakened and protracted limbs, non-weight bearing on limbs, a “tip-toe” walking pattern, and back hunching (94). Other castration studies have shown contrasting results relating to posture: more sitting and standing and less lying was seen in one study (91), while another observed increased lying and decreased standing (92). Torrey et al. (69) observed more standing and less lying in tail-docked, ear-notched, and sham-processed piglets compared to undisturbed controls, indicating that the alteration in posture was due to handling, regardless of the painful procedure. Other studies did not find differences in the time spent in different postures in relation to painful procedures (64, 75, 76, 93).

The frequencies of posture changes and durations of postural behavior have been used in assessing pain in sows arising from farrowing (95–97, 99–101), the presence of shoulder ulcers (73), and lameness (82, 90, 98). Naloxone (an opioid antagonist) administration increased standing, lying ventrally, and postural changes in periparturient sows, indicating that endogenous opioids may have a role in reducing pain at farrowing, which is manifest by frequent posture changes (101). In contrast, Lawrence et al. (102) recorded decreased frequency and duration of standing in naloxone-treated sows. Posture and the number of posture changes have been used to evaluate the use of post-farrowing analgesics (95–97, 100). Haussmann et al. (100) demonstrated that sows administered butophanol for 3 days post-parturition adopted fewer body position changes between 48 and 72 h afterward. Sows administered meloxicam for three consecutive days spent less time lying on the third day post-partum compared with placebo-treated animals (97). No difference in the amount of standing or duration of standing bouts was detected between sows given a single injection of meloxicam or placebo post-farrowing (95). For sows given ketoprofen for 3 days post-farrowing, no overall treatment differences in posture were observed, but younger sows treated with ketoprofen had more body position changes (96). Sows with shoulder ulcers spent less time lying and standing still and exhibited more posture changes than healthy animals (73).

Naturally lame sows treated with meloxicam showed an increase in standing time after feeding compared with those given a placebo (82). Predictably, sows rendered lame by intra-articular chemical irritant injection showed increased lying and decreased standing behavior (90, 98). Chemically induced lame sows treated with meloxicam lay down less frequently than saline-treated individuals, although those treated with flunixin did not differ from those in the placebo group (98).

#### Variation in Normal Behavior

Other behavioral alterations have been measured before and after painful events (64) and compared with control or sham animals (66, 70, 71, 73, 91–94, 103–105) from which analgesics were given or withheld (71, 72, 75–77, 79, 92, 103, 105, 106). Avoiding social contact with littermates has been recorded after tail docking (72) and castration (71, 93). Isolation, i.e., the state of being alone or with just one litter mate, and desynchronization, defined as activity that differs from 75% of littermates, was apparent up to 3 days following castration (70, 93). Other castration studies, however, have revealed no differences in social cohesion (75–77). The effects of painful procedures on teat seeking, udder mouthing/massaging, and nursing behavior are inconsistent. One study found castrated piglets spent more time massaging the udder (93), while others have shown a reduction in the duration of suckling in castrated individuals (92, 93, 103), longer latencies to participate in udder massage (64), or reduced levels of activity at the udder (77). In addition, sows with shoulder ulcers spent less time nursing than those without (73). Piglets castrated under azaperone/ketamine anesthesia used significantly more teats post-castration compared to pre-castration, which caused disturbance in suckling behavior (106). In addition Mcglone and Hellman (103) found that piglets given a general anesthetic missed sucklings, whereas no other piglets did. Both reports seem to suggest a detrimental effect of anesthesia on suckling activity. Other studies found no difference in suckling behavior in relation to castration (71, 76, 91, 92, 104) or tail docking (72).

Pain usually reduces feeding behavior in pigs. A significant reduction in feed intake and drinking behavior is associated with castration in 7- and 8-week-old pigs (92, 103). Feed intake for sows given meloxicam or a saline placebo post-farrowing was not statistically different (97), but feed refusal during lactation was delayed in sows given ketoprofen post-farrowing (107). Feed intake was used to assess the benefit of ketoprofen compared with flunixin or untreated grower pigs experimentally infected with *Actinobacillus pleuropneumoniae* (108), and altered feed intake (using a 4-point scale in which 1 indicated no change and 4 inappetance) was used as part of a clinical index score to assess the efficacy of meloxicam in the treatment of sows with mastitis–metritis–agalactia (MMA) (109). Food consumption decreased after thoracotomy surgery in pigs (110), and time spent feeding has been used to assess the efficacy of pain mitigation strategies in pigs undergoing abdominal surgery (111) and a femoral fracture model (112). In a study using a feed reward collection test to assess sows’ motivation to obtain feed rewards with different levels of lameness, moderately and severely lame sows obtained fewer rewards than mildly and non-lame sows (113). Unexpectedly, tail-bitten pigs, which had been given ketoprofen, spent less time at the feeder compared with those given a saline placebo (114).

Additional behavioral variables measured in relation to pain include aggression (70, 71, 77), playing, nosing, chewing and licking (64, 70, 75, 77, 93, 111), general activity (70, 72, 75, 76, 79, 91, 93, 94, 105, 106, 110), and location within the pen (76, 92, 103, 106). Painful procedures appear to disturb in particular interactive behaviors (chew, lick, play, nose, and aggression) (70, 77, 93, 104). Surprisingly, some studies have found no differences in general activity associated with tail docking and castration (72, 75, 76, 91). One report indicated that the location of piglets in relation to the heat lamp was altered following castration (103), while other studies found no differences (70, 76, 92). Castrated piglets took longer to navigate a handling chute at 0 and 15 min post-castration, with a reduction in navigation time observed only at 0 min after administration of preemptive meloxicam (105). A greater latency to move was measured in castrated compared with sham-castrated individuals when piglets were returned to the pen following the procedure (94). Reduced activity levels were returned to pretreatment levels 3 days after thoracotomy surgery (110), and latencies to perform active behaviors (rooting, eating, and drinking) were shorter for pigs given additional analgesia after abdominal surgery (111). The administration of buprenorphine increased the activity of lame pig in an open field test (79), although opioids in the absence of pain can increase activity levels.

#### Behavior Scores

Experimental studies of animal pain frequently score changes in both specified and unspecified behaviors to produce rating scales. Scoring systems include the visual analog scale (VAS), which is usually scored on a 10-cm line, numerical rating scales (e.g., 0–4), simple descriptive scales (e.g., mild, moderate, and severe), and variable rating scales, where several parameters are scored and the total of these used to indicate severity. For example, one study investigating castration pain used a variable rating scale – referred to as a Global Behavior Score (GBS), which combined scores for the presence of prostration, tremors, tail movement, and self-isolation (78). Following laryngeal transplantation, mini pigs were assessed for pain twice daily by wound palpation, behavior, and locomotion and given a score between 0 (no pain) and 10 (worst possible pain) (115). Two types of analgesia for post-thoracotomy were assessed in pigs (110) using the scoring system presented by Morton and Griffiths (15). Using a 4-point scale from 0 = no change to 3 = gross change from normal, patients were scored on five parameters including body weight, appearance, clinical signs, unprovoked behavior, and response to an appropriate stimulus, with the highest possible score being 15 (15). Two other studies of postoperative pain in pigs utilized a combination of scores for several spontaneous behavioral measures in addition to responses to pressure on the wound and human presence in the pen (116, 117). Behavioral responses of pigs undergoing surgery involving skin and muscle incision were greater compared to those with skin incision only at 1 h post-surgery (116). Another study used a behavioral score to assess the efficacy of regional anesthesia in addition to systemic opioid analgesia in a porcine femoral fracture model, which was a modified VAS with a scale for vocalization, behavior, on approach to the pen, response to handling, willingness to walk, and overall pain (no pain to worst pain imaginable) (112).

Pain scoring has been applied post-procedurally to pigs used in biomedical research to optimize postoperative care and assess differences in treatment methods. Evidence that an element of pain assessment is incorporated in these studies is good, albeit rare (5, 112). However, while these scores might be useful in a clinical setting, they may not be a valid approach to experimental pain assessment, as duration, frequency, or relative importance of individual behaviors is not considered. A more valid approach was demonstrated in a tail-docking study comparing docked with sham-docked piglets, by the use of a pain score constructed on behaviors recorded for 1 min immediately after docking (67). Combinations of all the behaviors recorded were analyzed in cross-validated discriminant analyses, which enabled the combination of behaviors that best discriminated between docked and sham-docked piglets to be used as part of the overall pain score.

Scores have also been used to assess lameness by the simple presence or absence of lameness (32, 118, 119), simple descriptive scales to indicate the varying degree of severity (120–123), and variable rating scales including several parameters relating to lameness (30, 124). A clinical lameness score, combining lameness at rest and at walk, was used to assess the efficacy of an intramuscular injection of meloxicam compared to a placebo to treat non-infectious locomotor disorders (122). Mustonen et al. (123) looked at two doses of oral ketoprofen compared to a placebo to treat non-infectious lameness, which they scored on a 5-point scale from no lameness to severe lameness. Both studies saw a significant improvement with drug treatment, compared with the placebo (122, 123). Nonetheless, some caution is needed, as locomotor disorders do not necessarily result in pain, with individuals possibly affected by a biomechanical abnormality in the absence of pain. Subjective scores are prone to poor reliability (125), and one study showed poorer interobserver agreement at the lower end of the lameness scale (126). For example, no difference in motivation to obtain a food reward was detected between mildly lame and non-lame sows (113). However, it is possible that pigs may conceal signs of mild pain more easily than signs of severe lameness-pain, which could be advantageous in relation to access to resources.

#### Quantitative Sensory Testing

Nociception defined as “*the neural processes of encoding and processing noxious stimuli*” (127) can be quantified in response to painful or potentially painful stimuli and is expressed as the minimum nociceptive threshold (i.e., latency or force/temperature) required to induce an avoidance response. Although QST only measures the sensitivity of the animals to detect a noxious stimulus, rather than pain, it allows the detection of abnormal thresholds caused by painful conditions, such as injuries or diseases in animals, including pigs. Jarvis et al. (128) measured the nociceptive threshold of pigs at pregnancy and parturition using a thermal stimulus provided by a CO_2_ infrared laser. The stimulation consisted of heat focused on the skin of sows, and it was interrupted with the occurrence of a physical response (e.g., a tail flick, movement of the back leg, or muscle twitch), providing an indication of the analgesic effect of endogenous opioids through detection of thresholds of sensitivity. A similar approach was used by Herskin et al. (129) to measure thermal nociceptive thresholds in two different anatomical locations on the pig (the back of the rear leg and the shoulder). The authors reported that increasing the power output of the laser decreased response latencies and altered the types of response from moving or lifting the leg to kicking, and from less obvious movement of the shoulder or body, to increased muscle twitching and rubbing of the area. Another nociceptive assay commonly used in laboratory species, the tail-withdrawal reflex induced by thermal stimulation, has also been applied to pigs. Immersion of the tail in warm water induced a tail flick, and response latencies were reduced by higher water temperatures (130). Other studies have used a modified version of the tail-withdrawal reflex test, using a plastic-coated electrical resistor, which is heated and pressed onto a selected area on the rump to induce a tail flick (131–133).

Mechanical nocistimulation have also found recent application in pigs. Herskin and Rasmussen (134) described thresholds of mechanical nociception in the pelvic limb of gilts using an electronic von Frey anesthesiometer, with four categories of behavioral response used to detect the threshold response, from slight leg movements to kicking. Sandercock et al. (135) measured mechanical thresholds at the tail root of pigs using von Frey filaments and at the foot with a plantar stimulator. The two methods were applied to investigate changes in thresholds of nociception with neonatal pain (tail docking) with or without prenatal stress. Tail docking did not result in any alterations in response to these tests but prenatally stressed pigs had higher thresholds to mechanical stimulation and longer response durations to cold stimulation. To determine the analgesic efficacy of ketoprofen in piglets, a handheld algometer was used to quantify mechanical thresholds pre- and posttreatment. The higher thresholds recorded in the ketoprofen-treated group compared to the placebo group suggested an anti-nociceptive effect between 12 and 24 h after drug administration, through the reduction of sensitivity of the peripheral nerve receptors (136).

The assessment of nociception threshold changes in pigs has been used in the study of traumatic pain – particularly lameness. In lame sows, a significant decrease in threshold values is revealed by pressure algometry (137, 138). The same methodologies have enabled researchers to assess the efficacy of NSAIDs in this context (80, 81). The assessment of nociceptive responses in pigs has received recent interest because their high homology with humans provides a translational model of induced cutaneous inflammatory pain. Methods for measuring thermal and mechanical nociceptive responses in awake pigs have been described, along with factors that may influence response thresholds (139–141). Models of cutaneous inflammatory pain, using capsaicin and UV-B irradiation have been used, demonstrating thermal and mechanical hyperalgesia in pigs (142, 143). Most recently, the choice of the pig as model for preclinical research has led to the characterization of postsurgical pain through the quantification of mechanical nociceptive thresholds (116, 144). Similarly, nociceptive stimuli delivered with an infrared diode laser have been employed to evoke withdrawal responses in the investigation of the efficacy of a novel analgesic (145).

#### Summary of Behavioral Assessment of Pain

To summarize the previous sections of discussion on behavioral indicators of pain in pigs, it is clear that spontaneous pain-related behaviors have most reliability in indicating pain across several studies. Some behavioral measures including posture, posture changes, altered social interaction, and exploration have demonstrated lower between-study reliability. Therefore, it seems behavioral indicators specific to the painful condition are the most reliable indicators of pain. The use of spontaneous pain-related behavior is not without problems; often the frequency of behavior is low and between-subject variation high, resulting in floor effects (83). Continuous behavioral observation is also time consuming, requires observer training, and is prone to interobserver variation (146). Therefore, specific behavioral indicators need to be carefully investigated and validated as pain indicators, as, for example, certain conditions involving physical injury or disease can result in loss of function, regardless of pain (125). In addition, behavioral expression of underlying welfare states has been shown to be altered by emotional contagion and social support, which could also be the case for pain (147–150). The social influence was mitigated in a tail-docking study, by observing pain-related behavior in piglets in isolation after the procedure (67); however, isolation in itself may alter the behavioral expression of pain (83). This time-consuming approach involving the detailed quantification and careful validation of behavior is useful in investigating pain in a research setting. However, the validation of complex behavioral patterns into simplified scores or rating scales, for the assessment and treatment of clinical pain on-farm and in biomedical research pigs, is much needed. Finally, QST is a useful method to investigate changes in peripheral nociceptive sensitivity as a consequence of injury or disease but does not address the multidimensional pain experience or how pain rather than peripheral sensitivity is ameliorated by analgesic treatment.

### Vocalization

Pigs also vocalize when painful areas are palpated but resent handling and may vocalize regardless of pain (27, 151). Several studies have found differences in the calls of piglets undergoing a painful procedure as opposed to handling alone, including call rate, duration, and type of call (64, 66, 75, 76, 78). More detailed analyses of calls using sound analysis software have revealed further differences in calls in relation to painful stimuli (88, 152–156). “Painful grunts,” along with persistent straining have been observed in cases of dystocia (27).

A higher grunt frequency was detected during tail docking compared to teeth clipping and ear notching, and howls were measured during tail docking but not the other two procedures (66). Sonograms of seven castrated piglets were analyzed, comparing them before and during castration: differences included a broader number of frequencies within the sound, a change of pitch, and a shift in volume during castration compared with the handling period (64). Hansson et al. (75) used a decibel meter during castration and recorded the highest intensity level (in decibels) of piglets castrated with and without a local anesthetic (lidocaine). Piglets with the local anesthetic produced calls with a significantly lower intensity than those without. In a more detailed vocal analysis during castration, White et al. (152) compared piglets castrated at different ages and with and without a local anesthetic (lidocaine), and segments of calls with the highest energy frequency (HEF in kilohertz) were used for analysis. HEF was significantly higher in piglets without anesthesia and increased with age, but the HEF was lower during severing of the spermatic cord (152), which is thought to be the most painful part of surgical castration (154). Other studies investigating vocalization during castration categorize vocalization based on call frequency, high calls being ≥1000 Hz and low calls ≤1000 Hz and found the rate (91, 153, 154) and total duration (156) of high calls is associated with castration, tail docking. and ear notching (69). Piglets castrated with no analgesia “screamed” significantly louder than those given local anesthetic or that were sham-castrated (156). Also, analyzing pain-related vocalization in piglets during castration and with and without a local anesthetic (lidocaine), Marx et al. (155) identified three different call types from spectrograms: grunts, squeals, and screams. The number of screams, but not the other two call types, were significantly more frequent for piglets castrated without anesthesia, indicating an increase in the rate of screams is a good indicator of pain. Screams were differentiated from squeals by sound parameters, including a lower peak and main frequency (155).

Several studies on vocalization in relation to pain use a device called STREMODO (STREss MOnitor and DOcumentation unit), which uses linear prediction analysis (157) to extract features of calls and classifies them as stress calls, as opposed to non-stress calls or background noise (158, 159). Puppe et al. (160) used STREMODO to detect stress calls of piglets undergoing castration and measured different vocal parameters. The surgical and postsurgical period differed in call duration, peak frequency, pureness, and entropy of sound, but not number of calls, and the surgical and presurgical period differed in the pureness and entropy of the sound. Therefore, during castration, the calls became longer and clearer with lower pitch. Leidig et al. (87) used STREMODO to detect stress calls during castration and compared the total duration of stress calls with piglets undergoing castration with or without a local anesthetic (procaine) or handling alone. Duration of stress calls increased from the pretreatment handling period to the surgical period, except for the sham animals, and was significantly longer in the castrated group without anesthesia but was also increased on the injection of anesthesia. Sutherland et al. (71, 72) used STREMODO to compare vocalization in relation to castration and tail docking, observing more stress vocalization with the painful procedures.

### Physiology

#### Neurotransmission

The expression of the c-fos gene and its protein product Fos in neurons of the spinal cord are used as a measure of neural activity in response to painful stimuli [for review, see Ref. (161)]. In pigs, the number of spinal Fos-positive neurons has been measured following castration with no anesthesia, a local anesthetic, or a general anesthetic (162) and following laparotomy with local anesthesia or a saline control (163). Following surgical castration with no anesthetic, a greater number of neurons in the dorsal horn were Fos positive, compared with piglets given a local or general anesthetic (162). Pigs that underwent experimental laparotomy with a local anesthetic as well as a general anesthetic had fewer Fos-like-immunoreactive neurons in the dorsal horn than those given saline and had a similar number to controls (163).

Substance P (SP) is a neurotransmitter released directly from damaged nerve fibers at the site of tissue damage (164, 165); it has been related to pain perception (166), making it a potential biomarker of pain. SP was elevated in compressed spinal nerve roots, compared with uncompressed controls, in a porcine model (166). Piglet castration did not elicit a plasma SP response at 3 days old (167) or in piglets that were castrated, tailed docked, and had iron administered at 5 days old (168). A study of castration indicated that SP could be a useful pain biomarker in calves (169), and SP has been shown to have a systemic role (170), indicating that measurement of systemic concentrations, rather than just at the site of damage, could be useful. However, further study is needed to indicate if this is a useful systemic biomarker of pain in pigs.

#### Cortisol and ACTH

Many studies have used measures of the activity of the hypothalamic–pituitary–adrenal (HPA) axis in relation to pain. The HPA axis is activated by physical and psychological stressors to promote recovery by increasing metabolism and reducing inflammation (21). In response to physical or psychological stress, corticotrophin-releasing hormone (CRH) is released by the hypothalamus, stimulating the secretion of adrenocorticotrophic hormone (ACTH) from the anterior pituitary, which acts on the adrenal gland to produce cortisol. Studies have investigated cortisol/ACTH levels in pigs with and without (controls/shams) a painful condition (68, 70, 72, 88, 93, 94, 104, 156, 171, 172), before, during, or after (68, 173–175), with or without anesthesia or analgesia (72, 78, 111, 156, 168), and associated with the severity of the pain (68, 88, 101, 171, 176).

In the periparturient period, sow corticoid levels increased from day 3 before parturition, peaking on the day of parturition and returning to pre-parturition levels on day 2 afterward (173). Cortisol and ACTH were higher during the pre-farrowing nest-building period in slatted crates compared to straw-bedded pens, indicating that restricting nest-building behavior is stressful (174, 175). However, after the onset of farrowing, no difference in levels of cortisol or ACTH between environment and the fact that these hormones remain elevated indicates that the process of parturition itself is stressful (173–175), and part of this could be pain related. Looking in more detail after the birth of the first piglet, cortisol concentration gradually increased with successive births (176) and was greater following the birth of larger piglets (101).

Studies have measured cortisol and/or ACTH in relation to tail docking (68, 72, 88, 171), teeth resection (88, 171), identification, iron administration (88), and castration (88, 94, 104, 156, 168, 171, 172). The majority of castration studies show an increase in cortisol and/or ACTH (88, 94, 104, 156, 171, 172). One study found no differences in cortisol and cortisone measured in urine 4 days following castration, sham castration, or piglets left undisturbed (70). The authors suggest that the sampling times may have been inadequate or that cortisol and cortisone in urine may not be good measures of sub-chronic and chronic pain. Cortisol has also been shown to increase with tail docking (68, 72), but not in all cases (68, 171), and no difference in cortisol between two different docking methods (side-cutter pliers or a hot cautery iron) was found (88). The authors of one study have four possible hypotheses for this lack of response that are as follows: (1) the HPA axis is not responsive to stress at 1-day-old; (2) it is highly responsive, and the maximum response is achieved by sampling; (3) the birth process; or (4) nociceptive stimuli from tail docking and teeth clipping is not enough to induce a response (171). For piglets that were ear-notched as opposed to tagged, cortisol tended to be higher 4 h after the procedure and for piglets with iron administered by injection rather than orally, cortisol was significantly higher 2 days after dosing (88). Cortisol tended to be higher 1 week post teeth grinding compared to clipping, but this difference disappeared with baseline values as a covariate (88), and another study showed an increase in cortisol and ACTH with teeth clipping and grinding up to 15 min following the procedures (171).

Piglets castrated with meloxicam administered before the procedure had lower cortisol and ACTH 30 min after compared to those given a saline placebo, and the meloxicam group had values of ACTH similar to controls (78). In addition, meloxicam administered *via* the transmammany route resulted in lower cortisol concentrations in castrated, tail-docked, and iron-injected piglets 10 h after the event (168). Another study found no differences in cortisol when castrated piglets were given local anesthetic, meloxicam, both, or no analgesia (156), and no difference in cortisol was found between tail docking with no anesthetic, topical anesthetic, or general anesthetic (72). Piglets castrated with or without anesthesia, had no difference in ACTH measured immediately afterward, despite obvious differences in other measures, and the authors suggest that handling or sampling had an effect or the collection time was inadequate (89). In an investigation of pain induced by abdominal surgery in pigs, epidural morphine was administered pre-op in combination with transdermal fentanyl patches post-op or a placebo of both. Pigs given the analgesia had significantly lower cortisol immediately after surgery, and levels decreased in both groups on the 3 days following surgery (111). In another study involving abdominal surgery, ACTH increased 15 min and remained significantly higher until 60 min post-surgery in pigs given a saline placebo in contrast to animals receiving local anesthesia, which showed no increase in ACTH (163).

#### Autonomic Responses

The sympathetic adrenomedullary (SA) system, part of the autonomic nervous system (ANS), is involved in mobilizing the body for “fight-or-flight,” increasing heart and respiration rates, paling or flushing, slowing or stopping digestion, constricting blood vessels, liberating nutrients (blood or glucose), dilating blood vessels for muscles, dilating pupils, etc. Skin temperature is thought to be a good indicator of SA response (75) as blood vessels in the skin constrict as blood is directed to the muscles and/or internal organs in preparation for fight-or-flight. Ear temperature was significantly higher for castrated piglets given no analgesia compared to those given a local anesthetic or a local anesthetic with an NSAID (75). By contrast, castrated, tail-docked and iron-injected piglets with transmammary-administered meloxicam had higher cranial temperature than those given a placebo (168). In another study, eye temperature was increased in castrated piglets up to 4 h after the procedure, rectal temperature was also greater at 3 and 5 h post-procedure, but glucose and lactate were not altered (94).

In a study investigating electroencephalographic and cardiovascular responses to nociception under isoflurane anesthesia, pinching of the nasal septum, anus, periople, and inter-digital skin and clamping of the tail and hind claw were used (177). Electroencephalographic measures and heart rate did not alter, but mean arterial blood pressure (MAP) increased with noxious stimulation, therefore was the most sensitive measure of nociception. Castration without local anesthetic resulted in a significantly greater rise in MAP and fall in pulse rate compared to piglets given an intratesticular or intrafunicular local anesthetic (178).

#### Endogenous Opioids

Beta-endorphin (β-end) is one of three families of opioid peptides, it is involved in regulating the body’s response to stress, including pain [for review on biology and function, see Ref. (179)]. The endogenous opioid β-end has been measured around parturition and is thought to be involved in reducing pain (176), maintaining passivity (180), and regulating oxytocin release (102). The administration of naloxone reduced the nociceptive threshold of sows, but not fully, indicating an endogenous opioid mechanism is perhaps only partly involved in hypoalgesia around parturition (128). Teeth resection by grinding rather than clipping produced a significantly higher β-end concentration 4 h after the procedure, and cutting the spermatic cord as opposed to tearing produced a higher β-end following castration (88). No difference in β-end concentrations was found between castrated piglets with isoflurane, isoflurane, and nitrous oxide general anesthetics or no anesthesia (89) and between pigs given epidural morphine before and fentanyl patches after or placebos when undergoing abdominal surgery (111). Alterations in β-end found by teeth grinding vs. clipping and cut vs. tear methods of castration were discussed by the authors and could be associated with other factors, including the increased blood loss from cutting and the increase in β-end with ACTH and cortisol being catabolic (88). Similarly, the lack of difference in β-end following castration with and without anesthesia or abdominal surgery with and without analgesia could be due to the stress of blood sampling producing a response regardless of treatment or inadequate sampling times (89, 111).

#### Immune Function

Inflammation, an immediate response to injury or infection, is characterized by redness, swelling, heat, pain, and diminished function. However, the magnitude of the inflammatory response and the pain experienced are not necessarily proportional (181). Inflammation is also associated with the acute-phase response, which causes an increase (positive) or decrease (negative) in acute-phase proteins (or APPs) triggered by pro-inflammatory cytokines from injured or infected cells. Concentrations of APPs, including haptoglobin (Hp), C-reactive protein (CRP), and serum amyloid A (SAA) can assess the level of inflammation and/or tissue damage in pigs (182). Cytokines and acute-phase proteins have been measured in relation to tail docking (183), castration (75, 78, 93), and teeth resection (184), and with the administration of NSAIDs in relation to *Streptococcus suis* infection (185) and parturition (107) in pigs.

In piglets left intact, or tail-docked by hot cautery or blunt trauma methods, no difference in CRP levels was present at 3 weeks, although at 7 weeks, CRP levels were significantly greater in the intact animals, being correlated with tail-biting injury scores (183). The use of meloxicam administered before castration reduced the level of Hp compared to untreated controls but not to levels that showed statistical significance (78) and in groups given meloxicam following castration, fewer piglets had high levels of SAA (75). Another castration study measuring cytokines [tumor necrosis factor-alpha (TNF-α) and interleukin 1 beta (IL1-β)] and acute-phase proteins (CRP, SAA, and Hp) found no difference between castrated pigs and those that were handled only (93). However, both cytokines increased in castrated and handled groups indicating handling or sampling alone could initiate pro-inflammatory responses and CRP and Hp or, more likely, they could have been elevated as both groups were also tail-docked, teeth-clipped, and ear-notched previously. On day 1 following teeth clipping, grinding, or left intact, no difference in levels of CRP or SAA were found; however, on day 29 following treatments, both APPs were significantly higher than on day 1, and CRP was higher in teeth-clipped compared to ground piglets (184). The authors suggest that the acute-phase response could have been initiated by the process of weaning, face lesions in piglets with teeth left intact, or mouth lesions in those teeth ground or clipped.

In a study that used an endotoxin challenge, CRP and Hp increased, but no difference between groups given meloxicam or a placebo was seen; however, thromboxane B_2_ (a vasoconstrictor and platelet aggregator) was higher in the placebo group (186). Measurement of acute-phase proteins following subcutaneous injection of live *Streptococcus suis* showed a correlation with clinical signs of infection including the development of fever and lameness (185). The biomarkers serum aspartate aminotransferase (AST) and creatine kinase (CK) and the APPs Hp and SAA were measured in relation to the use of a post-farrowing NSAID (107). An increase in AST, CK, and SAA was seen with parturition indicating tissue damage and inflammation associated with the process of parturition, and a greater increase in these biomarkers was demonstrated with the use of the NSAID, which the authors suggested is likely to be due to local tissue irritation from the intramuscular injection (107).

### Summary of Pain Assessment Measures

Perceived pain in non-verbal patients cannot be directly measured, or based on other measures, it can only be inferred (151). This means there is no “gold standard” for pain assessment in pigs. Since validation is based on the measurement of an indicator against a “gold standard” (19), pain indicators must be thoroughly evaluated using experimental studies. As information on this topic is rapidly growing, a thorough examination of the literature is warranted. As discussed, a wide range of behavioral indicators have been used in the assessment of pain in pigs, in addition to physiological measures as potential biomarkers of pain. The majority of studies used several measures simultaneously, with different experimental approaches to evaluate the potential value of these measures as indicators of pain. These approaches are used to test validity, reliability, and sensitivity, which are important if measures are to be used in the clinical assessment of pain to provide treatment, to ethically evaluate the cost of pain, and to test the efficacy of existing and novel analgesic drugs (for veterinary and human use). Table 1 lists the indicators by the broad categories outlined in this review, showing the counts of studies that used these indicators, whether they have been validated using the approaches outlined in the Section “Introduction,” and some brief advantages and disadvantages as pain indicators. As shown in Table 1, the assessment of spontaneous “pain-related” behavior is the most promising method of assessing pain in pigs. This approach has been used extensively in the rodent literature to assess pain in order to provide the best possible treatment and refine painful techniques [e.g., Ref. (146)]. For clinical use, it is also useful to combine measures into scores of multiple behaviors and clinical signs. Physiological indicators are prone to low specificity to pain (Table 1); however, using multiple measures, including behavior and physiology, is useful to fully assess the impact of a painful condition or event on the individual.

**Table 1:** **Summary of pain indicators used in experimental studies involving the assessment of pain in pigs and how they have been assessed in relation to pain using the following methods: (1) individuals studied before, during, and after a painful event; (2) comparing individuals thought to be in pain to controls or shams; (3) observing individuals with and without analgesia or anesthesia; and (4) observing whether indicators increase with the severity of pain, counts of studies using these measures, followed by some advantages and disadvantages in relation to validity, specificity, and reliability**.

Pain indicator category		Testing method				Counts of studies	Advantages	Disadvantages
		1	2	3	4			
Behavior	Spontaneous “pain-related”	Yes	Yes	Yes	Yes	23	Validated by several studies, specific to pain, and reliable with observer training	Validation needed for specific painful conditions
	Escape/avoidance	Yes	Yes	Yes	No	6	Validated by some studies and reliable with observer training	Low specificity – can occur with handling alone
	Posture or posture change	Yes	Yes	Yes	No	20	Validated by some studies and reliable with observer training	Low specificity – can be altered with factors other than pain
	Variation to normal	Yes	Yes	Yes	No	26	Validated by some studies and reliable with observer training	Low specificity – can be altered with factors other than pain
	Behavior scores	No	Yes	Yes	Yes	17	Validated by some studies, can be specific to pain, and reliable with observer training	Validation needed for specific painful conditions and only an instantaneous sampling point
	Quantitative sensory testing	Yes	Yes	Yes	Yes	18	Reliable with training and specific to the sensory perception of noxious stimuli	Low validity to pain – indicates sensory perception but not necessarily perceived pain

	Vocalization	Yes	Yes	Yes	Yes	18	Some vocal characteristics validated as pain indicators, reliable with analysis of vocal characteristics	Some vocal characteristics can occur in other situations, requires complex analysis

Physiology	Neurotransmission	No	Yes	Yes	No	4	Can be reliably quantified and specific to the neurotransmission of noxious stimuli	Low validity to multidimensional pain – indicates sensory processing but not necessarily perceived pain
	Cortisol/ACTH	Yes	Yes	Yes	Yes	19	Can be reliably quantified and has been validated in relation to painful conditions	Low specificity – can increase in relation to other factors and “ceiling effect” possible
	Autonomic	Yes	No	Yes	No	5	Can be reliably quantified and has been validated in relation to painful conditions	Low specificity – can alter in relation to other factors
	Endogenous opioids	Yes	No	Yes	Yes	5	Can be reliably quantified	Low validity and specificity – can alter in relation to other factors and little change with analgesia
	Immune function	Yes	Yes	Yes	Yes	8	Can be reliably quantified	Low validity and specificity – not directly related to perceived pain

## Potential Future Approaches to Pig Pain Assessment

Behavior is often used as a welfare indicator because of the difficulty in interpreting physiological indicators. According to Dawkins, “*the most obvious, least intrusive and potentially most powerful alternative of all [to assess welfare] is the animal’s behaviour*” (187). Its unobtrusiveness means it is the most frequently used measure in the assessment of pain in research studies involving pigs. Behaviors (including posture, gait, tremors, and restlessness) are used by pork producers in Victoria, Australia in the on-farm assessment of pain in pigs (188). However, the continued use of behavior in pig pain studies, involving the validation and simplification of behavioral assessment is needed to facilitate its application in the clinical assessment of pain. In addition, simplifying and standardizing behavioral approaches to pain assessment would assist in comparing measures across studies, to better indicate the reliability of these measures.

Simple behavioral responses to noxious stimuli, including those used in QST, address the ability to respond to noxious stimuli, but not necessarily the more complex, multidimensional experience that is pain (9). In addition, combining the use of QST, with spontaneous pain-related behavior to assess pain reduction strategies is suggested, since spontaneous pain is not always directly linked to hypersensitivity (189). Complex spontaneous behaviors probably reflect pain better than simple reflex responses, but there are other approaches that could be used. Drawing on the recent advances in clinical models of pain research, which focuses predominantly on laboratory rodent, a large body of novel methodological approaches can be adapted for use in pigs. Pigs can be readily trained to perform well in various cognitive tests or operant conditioning tasks (190), making them a good model for assessing the affective-motivational dimension of pain, including conditioned place preference, conditioned place aversion, self-section of analgesia, and motivational trade-offs, which have been developed in rodent models (83).

Facial grimace scaling is being validated in an increasing number of species and may prove useful in pigs. In an eye-tracking study of pain behavior in rabbits, it was found that observers focused on the rabbits’ facial features rather than other body areas (irrespective of the observer’s experience) (191). A facial grimace scale was first created for laboratory mice (192), and others have since been created for rats (193), rabbits (194), horses (195), and sheep (196, 197). A Piglet Grimace Scale (PGS) has been developed by experienced observers, who identified several facial action units (FAUs) from images of piglets pre- and post-tail docking and castration (198). However, only one FAU (orbital tightening) differed in tail-docked piglets, pre- and post-procedure, and no FAUs differed for castrated piglets.

Another approach is to look at tests that involve motivational trade-offs. A recent study using lame sows trained to retrieve food rewards (113) and another study investigating how long castrated piglets take to navigate a handling chute (105) go some way to investigating a motivational trade-off in relation to pain. This approach could be investigated further in pigs, adapting examples of behavioral tests used in rodent models of pain, such as the place escape/avoidance paradigm (PEAP) (17), the “Escape test” (199), or the facial reward/conflict paradigm (200).

## Concluding Remarks

This review focuses on measurements reported in experimental studies investigating pain in pigs. However, most await validation, and many require simplification for field application. The use of methodologies established in other species deserves examination in pigs. Tests aimed at investigating affective states have been recently developed in pigs (201–205) and could be used to better assess the severity of pain states associated with livestock production methods. In a recent survey of pig farmers and veterinarians, the majority of respondents disagreed with the statement: “*pigs are not as sensitive to pain as humans*” (31). Therefore, research is warranted into the causes and consequences of pain in livestock production to evaluate the trade-off between the cost of painful procedures and the longer term welfare benefit or improvement in product quality (1). The increasing use of pigs in biomedical research [e.g., Ref. (5)] is justified because of their similarity with humans. It follows that model refinement and external scientific validity are optimized when the model and the modeled are treated identically, and this includes the effective management of pain. The creation of more clinically applicable pain assessment methods is a prerequisite to the burgeoning use of pigs in translational research.

## Author Contributions

SI did the majority of the writing and communicated with the coauthors to coordinate the document editing. RC provided advice on the biomedical research input to the review article and performed significant edits to the document. PG wrote a large part of the quantitative sensory testing section and provided advice and edits on the whole document. KR provided significant advice on the document structure, content, and editing, as this document evolved to its current form.

## Conflict of Interest Statement

The authors declare that the research was conducted in the absence of any commercial or financial relationships that could be construed as a potential conflict of interest.
